# Curcumol Suppresses CCF-Mediated Hepatocyte Senescence Through Blocking LC3B–Lamin B1 Interaction in Alcoholic Fatty Liver Disease

**DOI:** 10.3389/fphar.2022.912825

**Published:** 2022-06-28

**Authors:** Xiaoyu Qi, Shuguo Zheng, Mingyue Ma, Naqi Lian, Hongting Wang, Lerong Chen, Anping Song, Chunfeng Lu, Shizhong Zheng, Huanhuan Jin

**Affiliations:** ^1^ Department of Pharmacology, School of Pharmacy, Wannan Medical College, Wuhu, China; ^2^ School of Medicine and Holistic Integrative Medicine, Nanjing University of Chinese Medicine, Nanjing, China; ^3^ School of Pharmacy, Nantong University, Nantong, China; ^4^ Jiangsu Key Laboratory for Pharmacology and Safety Evaluation of Chinese Materia Medica, School of Pharmacy, Nanjing University of Chinese Medicine, Nanjing, China

**Keywords:** curcumol, cellular senescence, cytoplasmic chromatin fragments, alcoholic fatty liver disease, LC3B–lamin B1

## Abstract

Recent studies indicated that hepatocyte senescence plays an important role in the development of alcoholic fatty liver disease (AFLD), suggesting that inhibition of hepatocyte senescence might be a potential strategy for AFLD treatment. The present study investigated the effect of curcumol, a component from the root of Rhizoma Curcumae, on hepatocyte senescence in AFLD and the underlying mechanisms implicated. The results showed that curcumol was able to reduce lipid deposition and injury in livers of ethanol liquid diet-fed mice and in ethanol-treated LO2 cells. Both *in vivo* and *in vitro* studies indicated that supplementation with curcumol effectively alleviated ethanol-induced cellular senescence as manifested by a decrease in senescence-associated β-galactosidase (SA-β-gal) activity, a downregulated expression of senescence-related markers p16 and p21, and dysfunction of the telomere and telomerase system. Consistently, treatment with curcumol led to a marked suppression of ethanol-induced formation of cytoplasmic chromatin fragments (CCF) and subsequent activation of cGAS-STING, resulting in a significant reduction in senescence-associated secretory phenotype (SASP)-related inflammatory factors’ secretion. Further studies indicated that curcumol’s inhibition of CCF formation might be derived from blocking the interaction of LC3B with lamin B1 and maintaining nuclear membrane integrity. Taken together, these results indicated that curcumol was capable of ameliorating AFLD through inhibition of hepatocyte senescence, which might be attributed to its blocking of LC3B and lamin B1 interaction and subsequent inactivation of the CCF-cGAS-STING pathway. These findings suggest a promising use of curcumol in the treatment of AFLD.

## Graphical Abstract

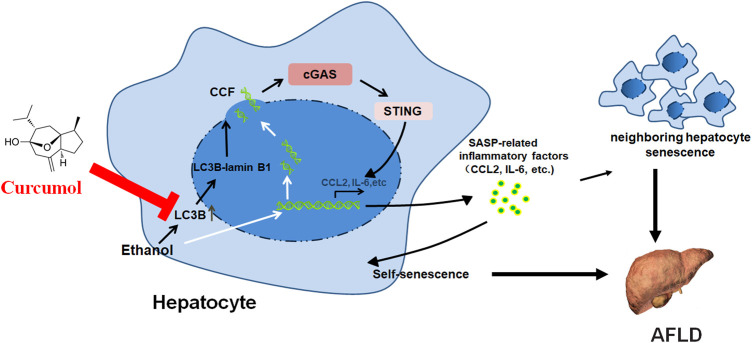

## Introduction

Alcoholic fatty liver disease (AFLD), a chronic liver disease characterized by excessive deposition of fat in hepatocytes, is caused mainly by chronic heavy alcohol consumption (>40 g of alcohol per day) over a sustained period (months or years) (Seitz et al., 2018; Caputo et al., 2019). Chronic exposure to alcohol could interfere with hepatic lipids metabolism through various mechanisms, including inhibition of mitochondrial β-oxidation and increase of fatty acid synthesis, resulting in a massive accumulation of triglycerides, phospholipids, and cholesterol esters in liver cells (Avila et al., 2020). Clinical data indicated that, if not intervened properly, AFLD would progress eventually to alcoholic hepatitis, liver fibrosis, liver cirrhosis, and even hepatocellular carcinoma (Addolorato et al., 2016; Wen et al., 2021). However, due to the fact that the exact mechanisms underlying the pathogenesis of AFLD remains unclear, there is still no effective method to reverse this disease process or prevent it from progressing to more severe stages.

Recently, several studies revealed that hepatocyte senescence participates in the development of alcoholic liver disease and suppression of hepatocyte senescence can effectively alleviate alcohol-induced hepatic steatosis, inflammation, and fibrosis, suggesting a potential strategy for the management of AFLD (Aravinthan et al., 2013; Jiang et al., 2021; Wu et al., 2021). Cellular senescence, a state of irreversible cell cycle arrest, is characterized by decreased proliferation capacity, increased senescence-associated β-galactosidase (SA-β-gal) activity, and senescence-related markers p16 and p21 expression, and the dysfunction of telomere and telomerase system (Collado et al., 2007; Hernandez-Segura et al., 2018). A growing body of evidence indicated that cellular senescence could be driven by various factors, including oxidative stress, inflammatory cytokines, and genotoxic stress (Bauer and Fuente, 2016), while the exact mechanisms remained unclear. Both *in vivo* and *in vitro* studies showed that alcohol could induce oxidative stress and subsequent double-strand DNA breaks (Gao et al., 2004; Abdelmegeed et al., 2013; Tete et al., 2020), which was considered a key player leading to hepatocyte senescence and AFLD. Meanwhile, most senescent cells can remain active for quite a long time and enter a state called senescence-associated secretory phenotype (SASP) (Malaquin et al., 2016). These senescent cells secrete numerous SASP-related inflammatory factors, including interleukin 6 (IL-6) (Shimizu et al., 2015), chemokine (C-C motif) ligand 2 (CCL2) (Zhao et al., 2019), colony-stimulating factor 2 (CSF2) (Huang et al., 2014), and vascular endothelial growth factor (VEGF) (Kumar et al., 2019), which can, in turn, accelerate the senescence of themselves and neighboring cells and form a vicious cycle (Sun et al., 2018; Kumari and Jat, 2021). However, it still remains unclear whether and how alcohol induces these factors’ secretion in hepatocytes.

Cyclic GMP-AMP synthase (cGAS), a cytosolic DNA sensor, can catalyze the formation of the second messenger 2′3′-cGAMP, which then binds to the stimulator of interferon genes (STING) and activates the NF-κB signaling pathway, resulting in an upregulation of a series of inflammatory factors, chemokines, and growth factors (Phelan et al., 2020). It has been proved that excessive activation of the cGAS-STING pathway is implicated in a number of inflammatory and autoimmune diseases (Gluck and Ablasser, 2019; Loo et al., 2020). On encountering various stress conditions, cells initiate the autophagy process, a homeostatic lysosome-dependent process, to eliminate damaged or dysfunctional cellular organelles. Cells in this process express a large amount of autophagy protein light chain 3 (LC3), a protein involved in autophagy membrane trafficking and substrate delivery, which can interact with the nuclear lamina protein lamin B1 and lead to the loss of nuclear membrane integrity (Dou et al., 2015). At the same time, injured cells incur DNA damage, and specific chromatin fragments leak into the cytoplasm through an impaired nuclear membrane, forming cytoplasmic chromatin fragments (CCF) and leading to the activation of the cGAS-STING pathway (Ivanov et al., 2013; Han et al., 2020). As a potent inducer of oxidative stress, alcohol has been proven to induce autophagy in hepatocytes, but whether and how alcohol induced the cGAS-STING pathway in hepatocytes remains to be elucidated.

Curcumol, an extract from the Curcumae Rhizoma roots, possesses a therapeutic potential for many diseases, including cancer, microbial infections, inflammation, and hepatic fibrosis (Jia et al., 2018; Wei et al., 2019; Hashem et al., 2021). However, there are few breakthroughs of curcumol in the therapeutic intervention of AFLD at present. Identification of the anti-AFLD effect of curcumol is urgently needed. We have demonstrated that curcumol reduced ethanol liquid diet-caused liver injury and lipid accumulation in mice through the inhibition of hepatocyte senescence, but the precise mechanisms related to its inhibition of hepatocytes senescence still remain unclear. The present study investigated the ameliorative effect of curcumol on AFLD and the underlying mechanisms from the aspect of regulating the CCF-cGAS-STING signaling pathway related to hepatocyte senescence.

## Materials and Methods

### Reagents and Antibodies

The following compounds and reagents were used in this study: curcumol (Herbpurify, Chengdu, China); SA-β-gal staining kit (Cell Signaling Technology, Danvers, MA, United States); Cell Counting Kit-8, BCA protein assay kit, nuclear and cytoplasmic protein extraction kit (Beyotime Biotechnology, Shanghai, China); Oil Red O (Sigma, St. Louis, MO, United States); DMEM (GIBCO BRL, NY, United States); fetal bovine serum (ExCell Bio, Shanghai, China); alanine aminotransferase (ALT), aspartate transaminase (AST), alkaline phosphatase (ALP), and lipid metabolism indexes (total cholesterol (T-CHO), triglyceride (TG), low-density lipoprotein cholesterol (LDL-C), and high-density lipoprotein cholesterol (HDL-C) detection kits (Nanjing Jiancheng Bioengineering Institute, Nanjing, China); Trizol reagent (Invitrogen™, Thermo Fisher Scientific, Waltham, MA, United States); Pierce co-immunoprecipitation (Co-IP) Kit (Thermo Scientific™, Thermo Fisher Scientific, Waltham, MA, United States); FastKing RT kit (with gDNase), SuperReal PreMix Plus (SYBR Green) (TIANGEN BIOTECH, Beijing, China). The following primary antibodies were used in this study: p16, H3K27me3, γ-H2AX, LC3B (Cell Signaling Technology, Danvers, MA, United States); cGAS (Santa Cruz Biotechnology, Santa Cruz, CA, United States); telomerase reverse transcriptase (TERT), telomeric repeat binding factors 1 (TRF1) (Bioss, Beijing, China); p21, telomeric repeat binding factors 2 (TRF2), Lamin B1, and STING, β-actin (ProteinTech Group, Chicago, IL, United States).

### Experimental Animal Procedures

All animal experimental procedures were approved by the institutional and local committee on the care and use of animals of Wannan Medical College (Wuhu, China) and consistent with the National Institutes of Health (United States) guidelines. Male C57BL/6J mice of 8 weeks (20–22 g body weight) were obtained from Changsha Tianqin Biotechnology Co., Ltd. and housed under specific pathogen-free conditions. Curcumol was dissolved in sterile saline. After 1 week of standard liquid diet adaptive feeding, 50 mice were randomly divided into five groups (*n* = 10): 1) control group, 2) alcohol group, 3) alcohol + curcumol (30 mg/kg) group, 4) alcohol + curcumol (45 mg/kg) group, and 5) alcohol + curcumol (60 mg/kg) group. All mice except those in the control group were given a transitional liquid diet with a ratio of Lieber-DeCarli liquid diet (Panyod et al., 2016)/pair-fed diet (1/2, 1/1, 2/1) every 2 days to adapt to the alcohol liquid diet for 6 days and were then switched to Lieber-DeCarli liquid diet (Trophic Animal Feed High-tech Co., Ltd., Nantong, China; TP4030D) containing 28% of calories from ethanol for 40 days. In addition, those in groups 3–5 were given daily curcumol (30, 45, and 60 mg/kg) by gavage from day 17 to day 46, with doses adjusted according to body weight monitored weekly. Mice in the control group were fed a control diet without ethanol (Trophic Animal Feed High-tech Co., Ltd., Nantong, China; TP4030C) all through the experiment. The intake of diet in different groups was checked daily to ensure isocaloric feeding. At the end of the experiment, mice were weighed and anesthetized with sodium pentobarbital, followed by blood samples collection from abdominal aorta and serum separation. A small portion of fresh liver was fixed in 10% formalin, and the rest was stored at −80°C for subsequent experiments.

The second batch of male C57BL/6J mice was used for *in vivo* validation. A total of 50 mice were randomly divided into 5 groups (*n* = 8): 1) control group, 2) empty vector group, 3) alcohol + empty vector group, 4) alcohol + empty vector + curcumol (45 mg/kg) group, and 5) alcohol + LC3B plasmid + curcumol (45 mg/kg) group. The process is the same as the aforementioned experiment, except that the mice in groups 2–5 were injected with 50 μL lentivirus-packaged empty vector or LC3B overexpression plasmid (1 × 10^9^ TU/ml, GenePharma, Shanghai, China) through the tail vein on day 17, 27, and 37. The LC3B lentiviral vector was constructed by cloning the amplified Mus Map1lc3b gene fragment into the LV5-EF-1a-GFP&Puro vector. The empty vector contained only the LV5-EF-1a-GFP&Puro vector. At the end of the experiment, all mice were anesthetized with sodium pentobarbital, and blood and tissue samples were collected as mentioned earlier.

### Cell Culture

Human normal liver cell line LO2 cells were purchased from the Cell Bank of Chinese Academy of Sciences (Shanghai, China). LO2 cells were cultured in DMEM medium, supplemented with 10% fetal bovine serum, 1% penicillin, and streptomycin in a humid atmosphere of 95% air and 5% CO_2_ at 37°C.

### Cell Counting Kit-8 Assay

LO2 cells cultured in 96-well plates were incubated with 100 mM ethanol and different concentrations of curcumol for 24 h. Cell viability was detected using CCK-8 kit assay kits according to the manufacturer’s instructions.

### Cell Transfection With cGAS and LC3B Overexpression Plasmid

LO2 cells were transfected with cGAS or LC3B overexpression plasmid (GenePharma, Shanghai, China) using UltraCruz^®^ Transfection Reagent. cGAS overexpression plasmid was constructed by cloning the amplified Mb21d1 (Human) gene fragment into the pEX-3 (pGCMV/MCS/Neo) vector. LC3B overexpression plasmid was constructed by cloning the amplified mCherry-EGFP-LC3B (Human) gene fragment into the pEX-3 (pGCMV/MCS/Neo) vector. The blank vector contained only the pEX-3 vector. In short, LO2 cells cultured in 6-well plates were rinsed 3 times with PBS and incubated with a transfection solution containing 1 μg of overexpression plasmid or blank vector and 4 μL of transfection reagent in each well for 6–8 h. After being washed with PBS and cultured in a fresh medium for 24 h, the cells were treated with ethanol and curcumol as described previously and then subjected to corresponding tests.

### 5-Ethynyl-2′-deoxyuridine Incorporation Assay

Propagation of cells was cytochemically detected according to the manufacturer’s instructions (C0071S, Beyotime, China). Briefly, LO2 cells cultured in confocal dishes were incubated with 100 mM ethanol and different concentrations of curcumol for 24 h. Then, LO2 cells were incubated with the EdU staining buffer for 2.5 h, fixed by 4% polyformaldehyde, and stained the nuclear with Hoechst. Laser confocal microscopy was used to observe and photograph the stained cells. EdU-positive cells were quantified from three randomly selected fields in each well by software ImageJ.

### Serum Biochemical Analysis

Serum levels of liver injury indexes (ALT, AST, and ALP) and lipid profiles (T-CHO, TG, LDL-C, and HDL-C) were detected by commercial assay kits according to the manufacturer’s protocol.

### Hematoxylin and Eosin Staining

Fresh liver tissues were fixed in 10% formalin solution and embedded in paraffin. Slices of 4 μm were deparaffinized and stained with hematoxylin and eosin routinely.

### Oil Red O Staining and SA-β-Gal Staining

The oil red O staining is used to observe lipid droplets, and SA-β-gal staining is used to observe cellular senescence. Liver tissues were frozen in optimal cutting temperature (OCT) compound were cut into sections of 8 μm thickness, and LO2 cells cultured in 24-well plates were treated with ethanol, curcumol, and other reagents as described in the figures. Oil red O staining solution needs to be filtered 4 times to remove impurities, and the pH value of SA-β-gal staining solution needs to be adjusted to 5.9–6.1. Other experimental steps were operated according to the kit manufacturer’s instructions. Total cell numbers and the stained cell numbers were counted from 3 fields per sample. SA-β-gal-positive cells were calculated as the percentage of positive cells per unit area.

### Immunofluorescence Staining

Frozen sections (8 μm) of mice livers and LO2 cells cultured in confocal dishes were fixed with 4% paraformaldehyde for 15 min, rinsed with PBS 3 times, and then blocked with blocking buffer (5% goat serum/0.3% Triton™ X-100/PBS) for 1 h, followed by incubation with the corresponding antibody at 4°C overnight. After incubation with the fluorescent secondary antibody for 2 h at room temperature on a shaker and rinsing with PBS, DAPI solution was added and incubated for 10 min. Fluorescence was observed by laser confocal microscopy.

### Real-Time Quantitative Polymerase Chain Reaction

Total RNA was isolated from liver tissues or LO2 cells using Trizol reagent. Two μg of total RNA was reverse transcribed by FastKing RT kit (with gDNase) using a thermal cycler (T-100, BioRad, United States), and Real-time PCR was detected by SuperReal PreMix Plus (SYBR Green) on StepOne Plus Real-time PCR systems (Applied Biosystems, Thermo Fisher Scientific, United States). The expression of target genes was normalized to the invariant control glyceraldehyde phosphate dehydrogenase (GAPDH). Statistical analyses were based on 2^−ΔΔCT^ method. The primers of genes are listed in Table 1.

**TABLE 1:** The primers used for determination of mRNA levels in mice and human hepatocytes.

Primer sequence (5′ to 3′)		
Gene	Forward	Reverse
CCL2 (mouse)	TCT​CTC​TTC​CTC​CAC​CAC​CA	CGT​TAA​CTG​CAT​CTG​GCT​GA
IL-6 (mouse)	GAC​AAA​GCC​AGA​GTC​CTT​CAG​A	TGT​GAC​TCC​AGC​TTA​TCT​CTT​GG
VEGF (mouse)	CGGGCCTCGGTTCCA	GCAGCCTGGGACCACTTG
CSF2 (mouse)	TTG​GGC​AGA​GCT​AGC​TTT​CAA	TGC​ACA​CAT​GTT​AGC​TTC​TTC​TC
GAPDH (mouse)	CAA​CTA​CAT​GGT​CTA​CAT​GTT​C	CGC​CAG​TAG​ACT​CCA​CGA​C
CCL2 (human)	TCA​AAC​TGA​AGC​TCG​CAC​TCT	GGC​ATT​GAT​TGC​ATC​TGG​C
IL-6 (human)	CCA​GGA​GCC​CAG​CTA​TGA​AC	CCC​AGG​GAG​AAG​GCA​ACT​G
VEGF (human)	CTT​GCC​TTG​CTG​CTC​TAC​C	CAC​ACA​GGA​TGG​CTT​GAA​G
CSF2 (human)	AAT​GTT​TGA​CCT​CCA​GGA​GCC	TCT​GGG​TTG​CAC​AGG​AAG​TT
GAPDH (human)	GTC​TCC​TCT​GAC​TTC​AAC​AGC​G	ACC​ACC​CTG​TTG​CTG​TAG​CCA​A

### Western Blot Assay

LO2 cells and liver tissues were homogenized in RIPA lysis buffer (containing 1% protease inhibitor PMSF) on ice for 30 min. The supernatant was collected by centrifugation, and the protein concentration was measured by a BCA protein assay kit. Proteins were separated by SDS-PAGE and then transferred to the PVDF membrane. Following blocking with 5% milk for 2 h and incubation with primary antibody at 4°C overnight, the membranes were washed three times with TBST and incubated with horseradish peroxidase-conjugated secondary antibody at room temperature for 2 h. The results were observed by using an ECL detection kit (Millipore, WBKLS0500, Burlington, MA, United States), and protein levels were normalized to the invariant control β-actin or the total histone. The expressions of target protein bands were accurately determined by using Image Lab (NIH, Bethesda, MD) or ImageJ.

### Co-Immunoprecipitation Assay

First, the lamin B1 and the negative control IgG antibody were each fully absorbed by AminoLink coupling resin. Second, LO2 cells were lysed on ice for 5 min with the exclusive lysis buffer. After centrifugation at 13,000 g for 15 min, the supernatant was collected and the protein concentration was evaluated by a BCA protein assay kit. Then an appropriate amount of supernatant was added to each resin column, inverted, and mixed completely for 2 h at 4°C. The eluate was collected, mixed fully with 5* loading buffer and boiled for 5 min. The combination of Lamin B1 and LC3B was observed by western blot.

### Nuclear and Cytoplasmic Protein Extraction Assay

LO2 cells cultured in a 6-well plate were washed three times with PBS, scraped off and collected after centrifugation. Following vortex with 200 μL of reagent A containing 1% PMSF for 5 s, the cells were placed on ice for 10 min and then vortexed again with 10 μL of reagent B for 5 s. After incubation on ice for 1 min, the cells were vortexed vigorously for 5 s and subjected to centrifugation at 12,000 g for 5 min at 4°C. Cytoplasmic protein in the supernatant was collected immediately. Nuclear protein in the precipitate was extracted with a 50 μL nuclear extraction reagent according to the manufacturer’s instructions. All the proteins were determined by Western blot analysis.

### Statistical Analysis

The results were presented as mean ± SD, and the differences between groups were analyzed using GraphPad Prism 7.0 (GraphPad Software, San Diego, CA, United States). The significance of the difference was determined *via* a one-way analysis of variance with the post hoc Dunnett’s test. Values of *p* < 0.05 were considered statistically significant.

## Results

### Curcumol Ameliorated AFLD Through the Inhibition of SASP-Related Inflammatory Factors’ Secretion and Hepatocyte Senescence

**FIGURE 1:**
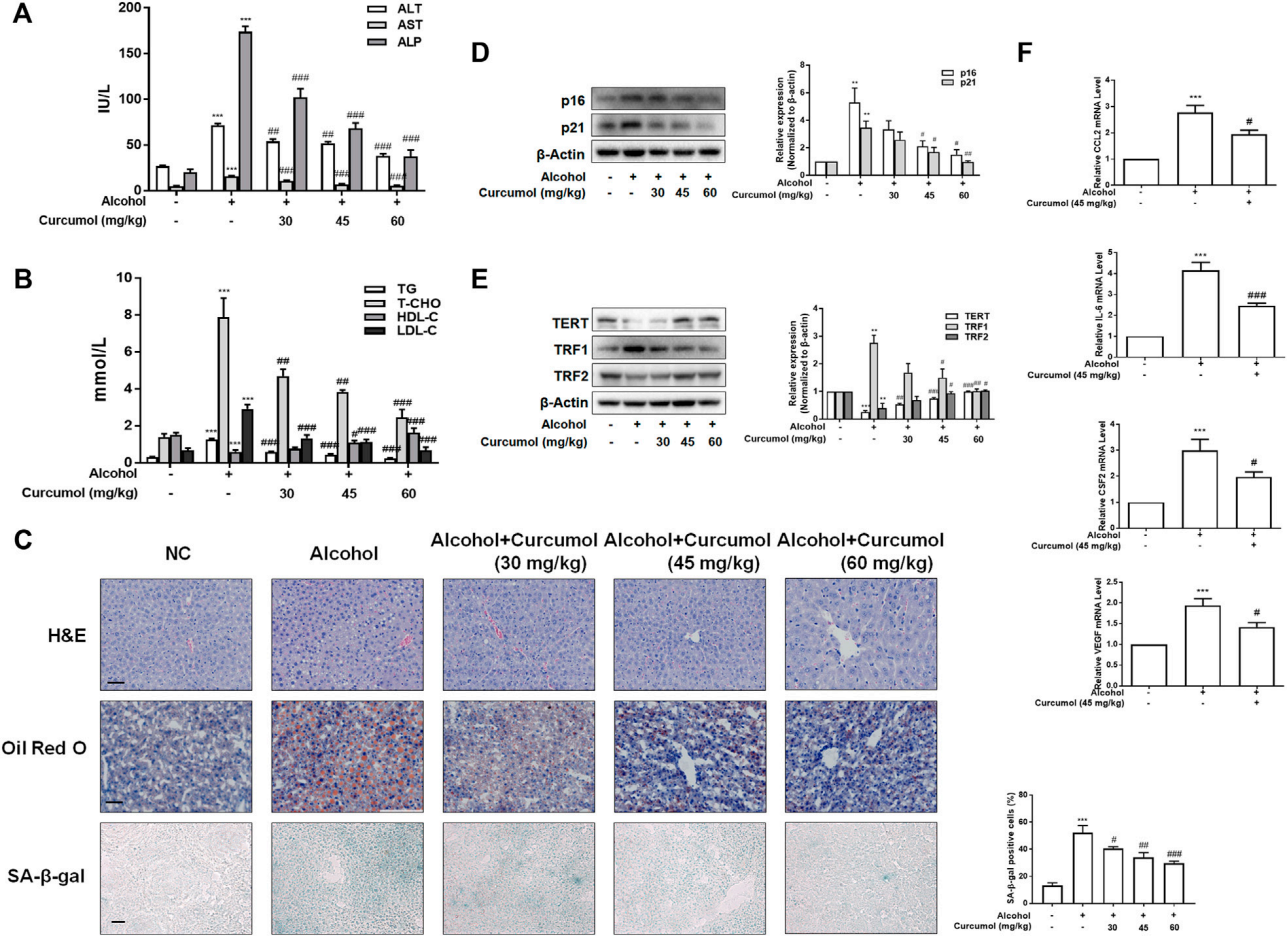
Curcumol ameliorates AFLD through the inhibition of SASP-related factors section and hepatocyte senescence. Male C57BL/6 J mice were fed with Lieber-DeCarli lipid diet in the model group and curcumol group while the control group was fed with a control diet without ethanol. **(A** and **B)** Determination of serum ALT, AST, ALP, T-CHO, TG, LDL-C, and HDL-C levels, *n* = 6. Data are represented as mean ± SD. Significance: ***p < 0.001 versus control group, ^#^ *p* < 0.05, ^##^ *p* < 0.01, and ^###^ *p* < 0.001 versus alcohol group. **(C)** Liver sections were stained with H&E, oil red O staining (scale bars, 50 μm), and SA-β-gal staining (scale bars, 100 μm), *n* = 3. Data are represented as mean ± SD. Significance: ***p < 0.001 versus control group, ^#^ *p* < 0.05, ^##^ *p* < 0.01, and ^###^ *p* < 0.001 versus alcohol group. **(D** and **E)** Western blot analyses of senescence markers (p16 and p21) and telomere and telomerase related factors (TERT, TRF1, and TRF2) protein levels in the liver tissues, *n* = 3. Data are represented as mean ± SD. Significance: **p < 0.01 and ***p < 0.001 versus control group, ^#^ *p* < 0.05, ^##^ *p* < 0.01, and ^###^ *p* < 0.001 versus alcohol group. **(F)** Real-time PCR analyses of hepatic inflammatory cytokines mRNA abundance, including CCL2, IL-6, CSF2, and VEGF, *n* = 3. Data are represented as mean ± SD. Significance: ***p < 0.001 versus control group, ^#^ *p* < 0.05 and ^###^ *p* < 0.001 versus alcohol group.

We established the AFLD model with Lieber-DeCarli ethanol liquid diet in C57BL/6J mice to investigate the protective effect of curcumol on ethanol-induced liver injury. The results showed that curcumol concentration-dependently decreased serum levels of ALT, AST, ALP, T-CHO, TG, and LDL-C and increased the level of HDL-C in mice with AFLD (Figures 1A and B). H&E staining and oil red O staining showed that curcumol alleviated ethanol-induced liver damage, including disorganized hepatocytes, loss of hepatic lobule structure and lipid accumulation (Figure 1C). We next detected the effect of curcumol on hepatocyte senescence in AFLD. SA-β-gal staining and western blot analyses showed that ethanol lipid diet increased hepatocyte senescence in mice, accompanied by a significant upregulation of protein levels of p16, p21, TRF1 and downregulation of protein levels of TERT, TRF2 in the liver of AFLD mice. Supplementation with curcumol effectively decreased the number of senescent hepatocytes, with above mentioned senescence-related protein levels reversed significantly (Figures 1D and E). Furthermore, we detected the mRNA levels of SASP-related inflammatory factors in mice liver tissue. The results showed that ethanol enhanced the expression of inflammatory cytokines such as CCL2, IL-6, CSF2, and VEGF, while supplementation with curcumol evidently inhibited the expression of these inflammatory cytokines. (Figure 1F). Taken together, these results indicated that curcumol could alleviate ethanol-induced liver damage in mice, which might be related to its inhibition of SASP-related inflammatory factors secretion and hepatocyte senescence.

**FIGURE 2:**
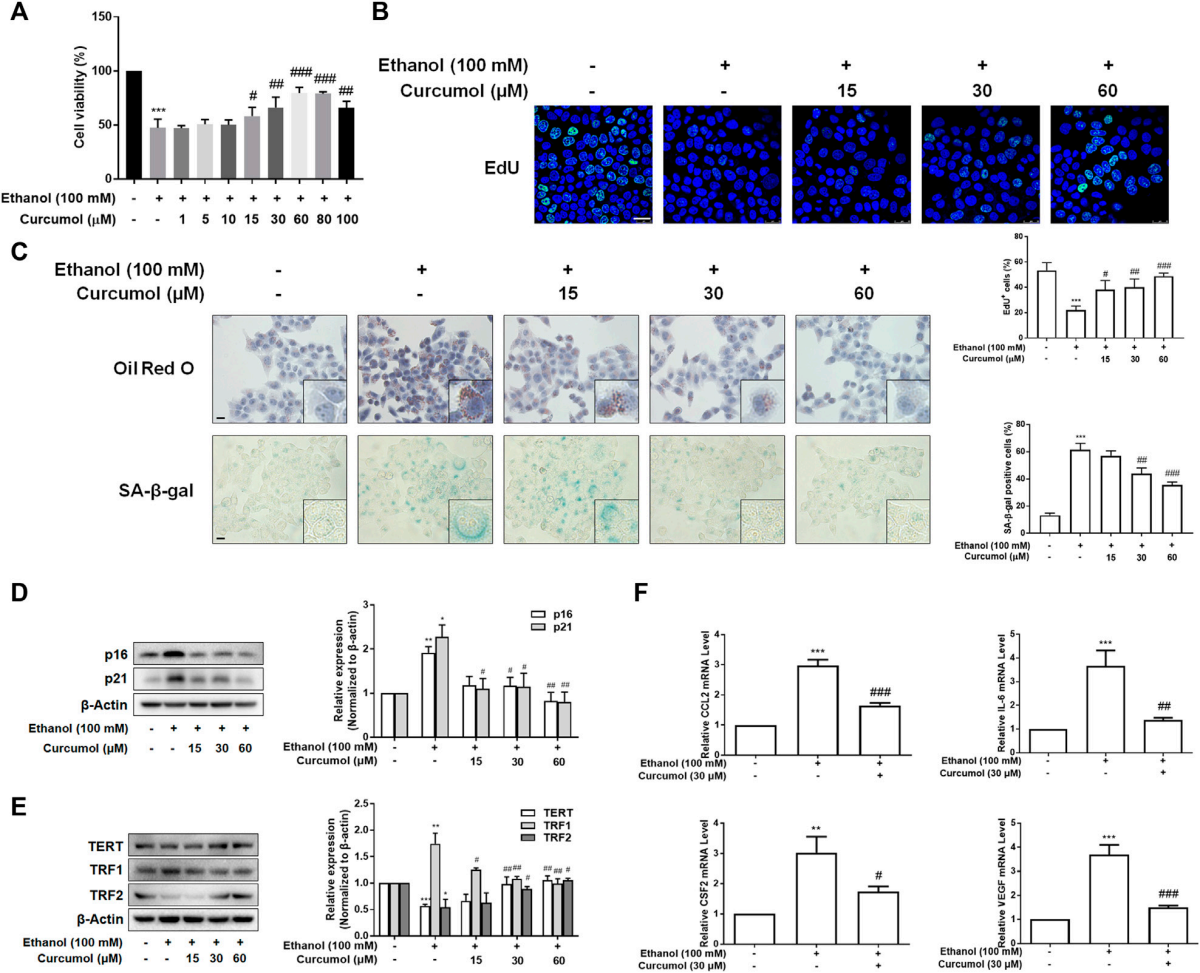
Curcumol inhibited hepatocyte injury and senescence, which may be related to the inhibition of SASP factor secretion. When LO2 cells grew to 70%–80% confluence, cells were incubated with 100 mM ethanol and different concentrations of curcumol for 24 h. **(A)** Cell Count Kit-8 analysis of cell viability, *n* = 6. Data are represented as mean ± SD. Significance: ^***^ *p* < 0.001 versus control group, ^#^ *p* < 0.05, ^##^ *p* < 0.01, and ^###^ *p* < 0.001 versus ethanol group. **(B)** The representative images and quantification of EdU-positive cells in LO2 cells under indicated conditions (*n* = 3). Scale bar, 25 μm ***p < 0.001 versus control group, ^#^ *p* < 0.05, ^##^ *p* < 0.01, and ^###^ *p* < 0.001 versus ethanol group. **(C)** Representative images of oil red O staining and SA-β-gal staining of LO2 cells, *n* = 3. Scale bar, 100 μm. Data are represented as mean ± SD. Significance: ***p < 0.001 versus control group, ^##^ *p* < 0.01 and ^###^ *p* < 0.001 versus ethanol group. **(D** and **E)** Western blot analyses of protein expression of senescence markers (p16 and p21) and telomere and telomerase related factors (TERT, TRF1, and TRF2) protein levels in LO2 cells, *n* = 3. Data are represented as mean ± SD. Significance: *p < 0.05, **p < 0.01, and ***p < 0.001 versus control group, ^#^ *p* < 0.05 and ^##^ *p* < 0.01 versus ethanol group. **(F)** Real-time PCR analyses of inflammatory cytokines mRNA abundance in LO2 cells, including CCL2, IL-6, CSF2, and VEGF, *n* = 3. Data are represented as mean ± SD. Significance: **p < 0.01 and ^***^ *p* < 0.001 versus control group, ^#^ *p* < 0.05, ^##^ *p* < 0.01, and ^###^ *p* < 0.001 versus ethanol group.

To further validate the effect of curcumol on ethanol-induced hepatocyte senescence, LO2 cells were treated with 100 mM ethanol to build an *in vitro* model of AFLD. The results of the CCK-8 assay showed that curcumol was capable of ameliorating ethanol-induced cell damage. However, when the concentration of curcumol reached 80 μM, cell viability began to decrease significantly (Figure 2A). Therefore, the following experiments were carried out with 15, 30, and 60 μM of curcumol. The analysis of cell proliferation revealed that the proportion of EdU-positive cells was lower in the ethanol group, which was remarkably increased in a concentration-dependent manner in the presence of curcumol (Figure 2B). As shown in Figure 2C, curcumol effectively suppressed ethanol-induced lipid accumulation and cellular senescence in LO2 cells, with the expression of senescence indicators mentioned above reversed evidently (Figures 2D and E). In addition, treatment with curcumol markedly suppressed ethanol-induced expression of inflammatory factors (Figure 2F). These results were consistent with the *in vivo* findings, providing further evidence for the ameliorative effect of curcumol on ethanol-induced hepatocyte injury and senescence.

### Curcumol Reduced Ethanol-Induced CCF Formation

**FIGURE 3:**
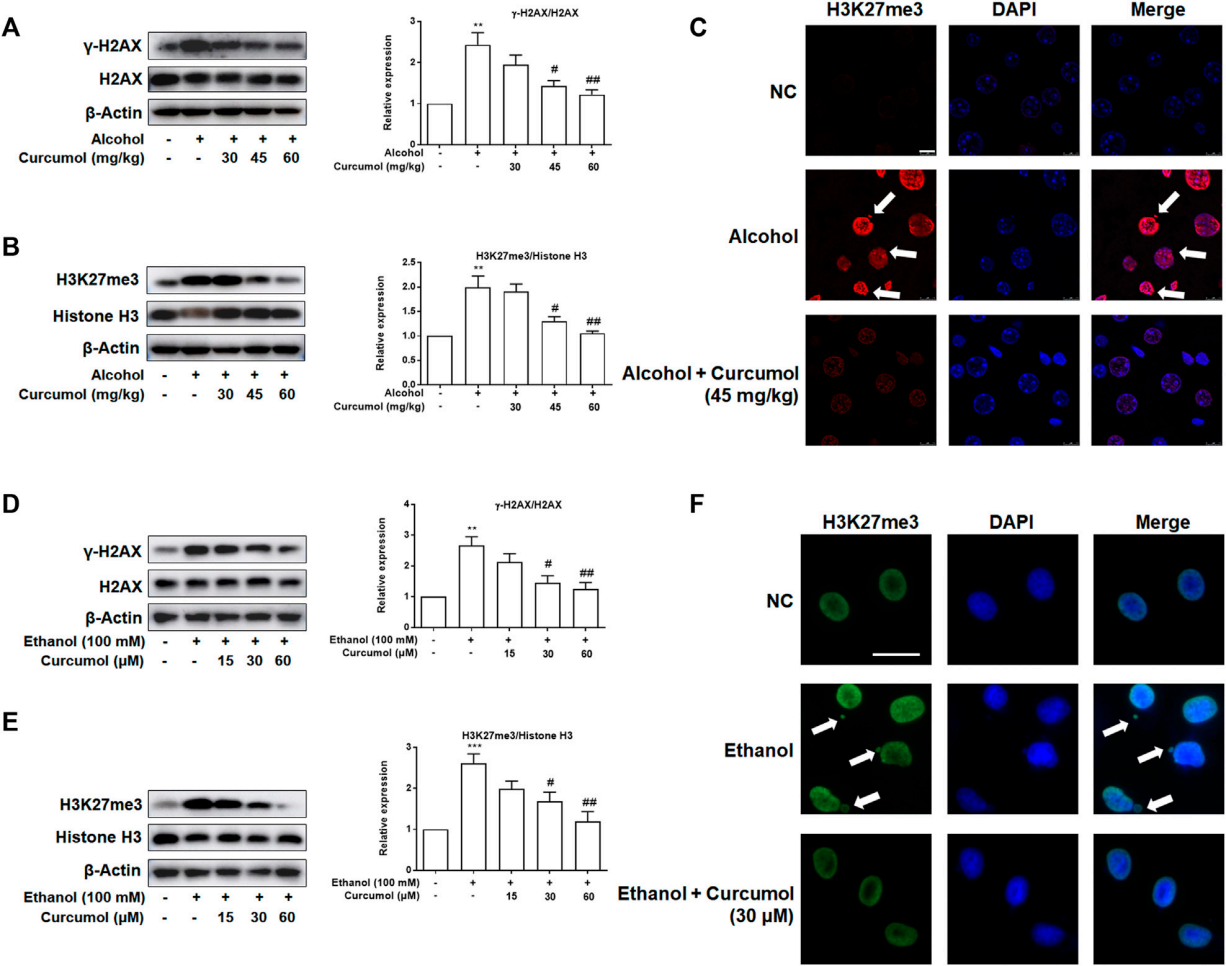
Curcumol reduced ethanol-induced CCF formation. **(A** and **B)** Western blot analyses of the classical markers of CCF (γ-H2AX and H3K27me3) protein levels *in vivo*, *n* = 3. Data are represented as mean ± SD. Significance: **p < 0.01 versus control group, ^#^ *p* < 0.05 and ^##^ *p* < 0.01 versus alcohol group. **(C** and **F)** Representative images of immunofluorescence analyses of H3K27me3 in mice’s liver tissues (scare bar, 7.5 μm) and LO2 cells (scare bar, 50 μm). CCF are marked with arrows, *n* = 3. **(D** and **E)** Western blot analyses of the classical markers of CCF (γ-H2AX and H3K27me3) protein levels *in vitro*, *n* = 3. Data are represented as mean ± SD. Significance: **p < 0.01 and ***p < 0.001 versus control group, ^#^ *p* < 0.05 and ^##^ *p* < 0.01 versus ethanol group.

Given that CCF is highlighted as a target molecule controlling cellular senescence in recent reports, we explored the effect of curcumol on ethanol-induced CCF formation in mice and cultured LO2 cells. Western blot analyses showed that curcumol dose-dependently decreased ethanol-induced γ-H2AX and H3K27me3, the classical markers of CCF, in the liver of AFLD mice (Figures 3A and B). Further studies by immunofluorescence staining demonstrated that there existed only a little nucleus-located H3K27me3 in mice liver of the control group, while in the liver of ethanol containing diet-fed mice, a large amount of H3K27me3 could be observed in the nucleus and ethanol treatment could make part of heterochromatin in the nucleus leaked into the cytoplasm to become CCF. However, this phenomenon was depressed by supplementation with curcumol (Figure 3C). The *in vitro* experiments performed with LO2 cells yielded similar results as in the *in vivo* studies (Figures 3D–F), providing further support for the inhibitory effect of curcumol on CCF formation in AFLD.

### The Activation of cGAS-STING Weakened the Effect of Curcumol on Cellular Senescence in Ethanol-Treated LO2 Cells

**FIGURE 4:**
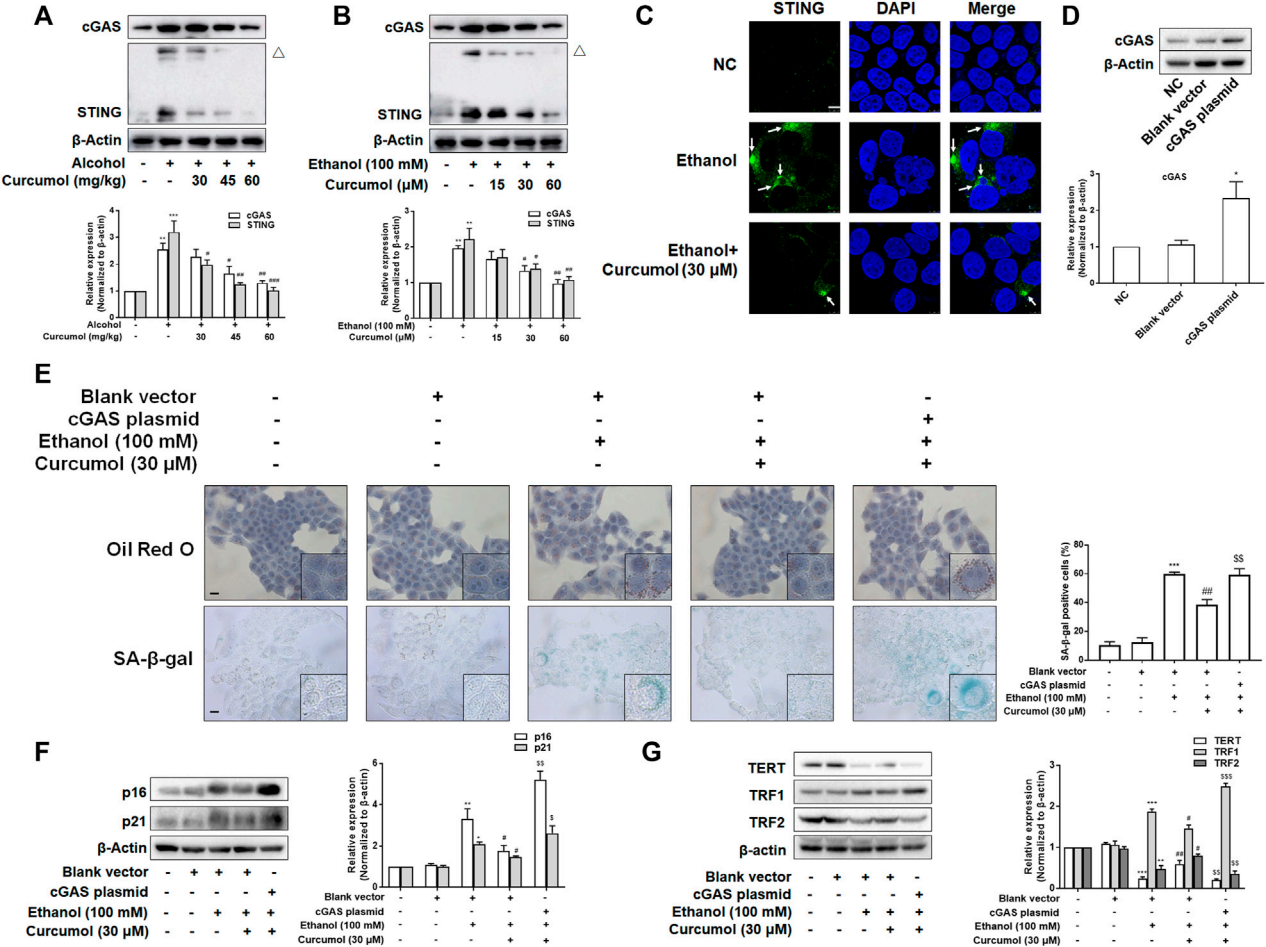
The activation of cGAS-STING weakened the effect of curcumol on cellular senescence in ethanol-treated LO2 cells. **(A)** Western blot analyses of the expressions of cGAS and STING *in vivo*, *n* = 3. STING blots were performed under non-reducing conditions. △ STING dimer. Data are represented as mean ± SD. Significance: **p < 0.01, and ***p < 0.001 versus control group, ^#^ *p* < 0.05, ^##^ *p* < 0.01, and ^###^ *p* < 0.001 versus alcohol group. **(B)** Western blot analyses of the expressions of cGAS and STING *in vitro*, *n* = 3. STING blots were performed under non-reducing conditions. △ STING dimer. Data are represented as mean ± SD. Significance: **p < 0.01 versus control group, ^#^ *p* < 0.05 and ^##^ *p* < 0.01 versus ethanol group. **(C)** Confocal microscopy images of STING in LO2 cells. Arrows are used to mark the redistribution of STING into aggregates. Scare bar, 7.5 μm. **(D)** Western blot analyses the cGAS plasmid transfection efficiency, *n* = 3. Data are represented as mean ± SD. Significance: ***p < 0.001 versus blank vector group. **(E)** Representative images of oil red O and SA-β-gal in LO2 cells, *n* = 3. Scale bar, 100 μm data are represented as mean ± SD. Significance: ***p < 0.001 versus blank vector group, ^##^ *p* < 0.01 versus blank vector + ethanol group, ^$$^ *p* < 0.01 versus blank vector + ethanol + curcumol group. **(F** and **G)** Western blot analyses of protein expression of senescence markers (p16 and p21) and telomere and telomerase related factors (TERT, TRF1, and TRF2) protein levels in LO2 cells, *n* = 3. Data are represented as mean ± SD. Significance: *p < 0.05, **p < 0.01, and ***p < 0.001 versus blank vector group, ^#^ *p* < 0.05 and ^##^ *p* < 0.01 versus blank vector + ethanol group, ^$^ *p* < 0.05, ^$$^ *p* < 0.01, and ^$$$^ *p* < 0.001 versus blank vector + ethanol + curcumol group.

Since the activation of the cGAS-STING pathway plays a pivotal role in mediating CCF-induced cellular senescence through promoting the massive release of SASP-related inflammatory factors, we investigated whether this pathway participates in hepatocyte senescence in AFLD and is necessary for curcumol’s inhibition of ethanol-induced hepatocyte senescence. Western blot analyses showed that the expression of cGAS and STING was largely increased in AFLD mice liver and ethanol-treated LO2 cells and was decreased by curcumol in a dose-dependent manner (Figures 4A and B). Curcumol inhibited the hallmarks of STING activation in AFLD mice liver and ethanol-treated LO2 cells, including the formation of homo-dimers (Figures 4A and B) and redistribution into aggregates (Figure 4C). Then, we transfected LO2 cells with cGAS plasmid, and the transfection efficiency was confirmed by western blot (Figure 4D). Overexpression of cGAS attenuated the ameliorative effects of curcumol on lipid accumulation and cellular senescence in ethanol-treated LO2 cells (Figure 4E). Further experiments indicated that curcumol’s effect on the expression of senescence-related markers and the telomere/telomerase system was also counteracted by cGAS overexpression (Figures 4F and G). These results indicated that curcumol could reduce ethanol-induced hepatocyte senescence through inhibiting the CCF-cGAS-STING pathway.

### Curcumol Inhibited LC3B Expression and Blocked LC3B–Lamin B1 Interaction

**FIGURE 5:**
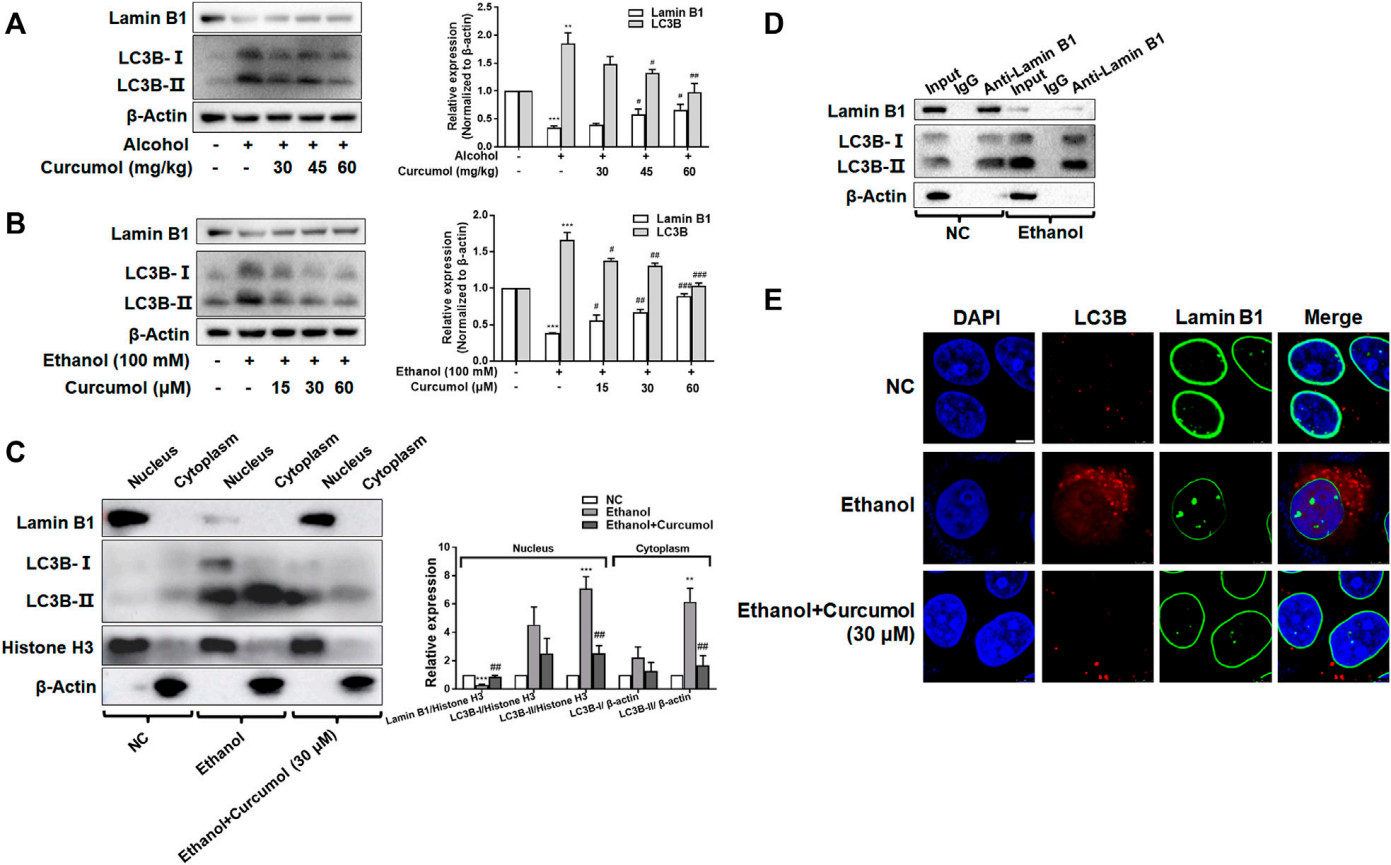
Curcumol inhibited LC3B expression and blocked LC3B-lamin B1 interaction. **(A)** Western blot analyses of protein expression of Lamin B1 and LC3B *in vivo*, *n* = 3. Data are represented as mean ± SD. Significance: **p < 0.01 and ***p < 0.001 versus control group, ^#^ *p* < 0.05 and ^##^ *p* < 0.01 versus alcohol group. **(B)** Western blot analyses of protein expression of Lamin B1 and LC3B *in vitro*, *n* = 3. Data are represented as mean ± SD. Significance: ***p < 0.001 versus control group, ^#^ *p* < 0.05, ^##^ *p* < 0.01 and ^###^ *p* < 0.001 versus ethanol group. **(C)** Western blot analyses of protein abundance of Lamin B1 and LC3B in the cytoplasm and nucleus, respectively, *n* = 3. Data are represented as mean ± SD. Significance: **p < 0.01 and ***p < 0.001 versus negative control group, ^##^ *p* < 0.01 versus ethanol group. **(D)** Co-IP analysis of the relevance between Lamin B1 and LC3B. **(E)** Immunofluorescence staining analysis of the localization of Lamin B1 and LC3B in LO2 cells, *n* = 3. Scare bar, 7.5 μm.

Nucleophagy can cause the loss of nuclear membrane integrity and result in CCF formation. However, whether ethanol-induced CCF formation is derived from nucleophagy and how curcumol reduced CCF formation in AFLD remained unclear. The results of both *in vivo* and *in vitro* studies showed that exposure to ethanol induced a significant upregulation of autophagy marker protein LC3B and downregulation of nuclear envelope protein lamin B1, and these changes could be significantly reversed by treatment with curcumol (Figures 5A and B). Further studies revealed that the increased LC3B (mainly LC3B-II) was located mainly in the nucleus under the treatment of ethanol, while curcumol could reduce the accumulation of LC3B in the nucleus (Figure 5C), suggesting that curcumol could inhibit nucleophagy in ethanol-treated LO2 cells, during which LC3B was unable to interact with lamin B1 to suppress lamin B1 degradation and nuclear envelope rupture. In order to further test this possibility, we used co-immunoprecipitation experiments to detect the interaction of LC3B and lamin B1 (Figure 5D). The results showed that the interaction of lamin B1 and LC3B existed in LO2 cells and ethanol treatment promoted the massive degradation of lamin B1. Furthermore, the result of immunofluorescence staining also verified the above conclusion (Figure 5E). These data collectively implied that ethanol caused CCF formation might be attributed to its induction of nucleophagy, and curcumol’s inhibition of CCF formation might be derived from its interference with LC3B expression and subsequent interaction with lamin B1.

### Overexpression of LC3B Attenuated Curcumol’s Effect on Ethanol-Induced Cellular Senescence in LO2 Cells

**FIGURE 6:**
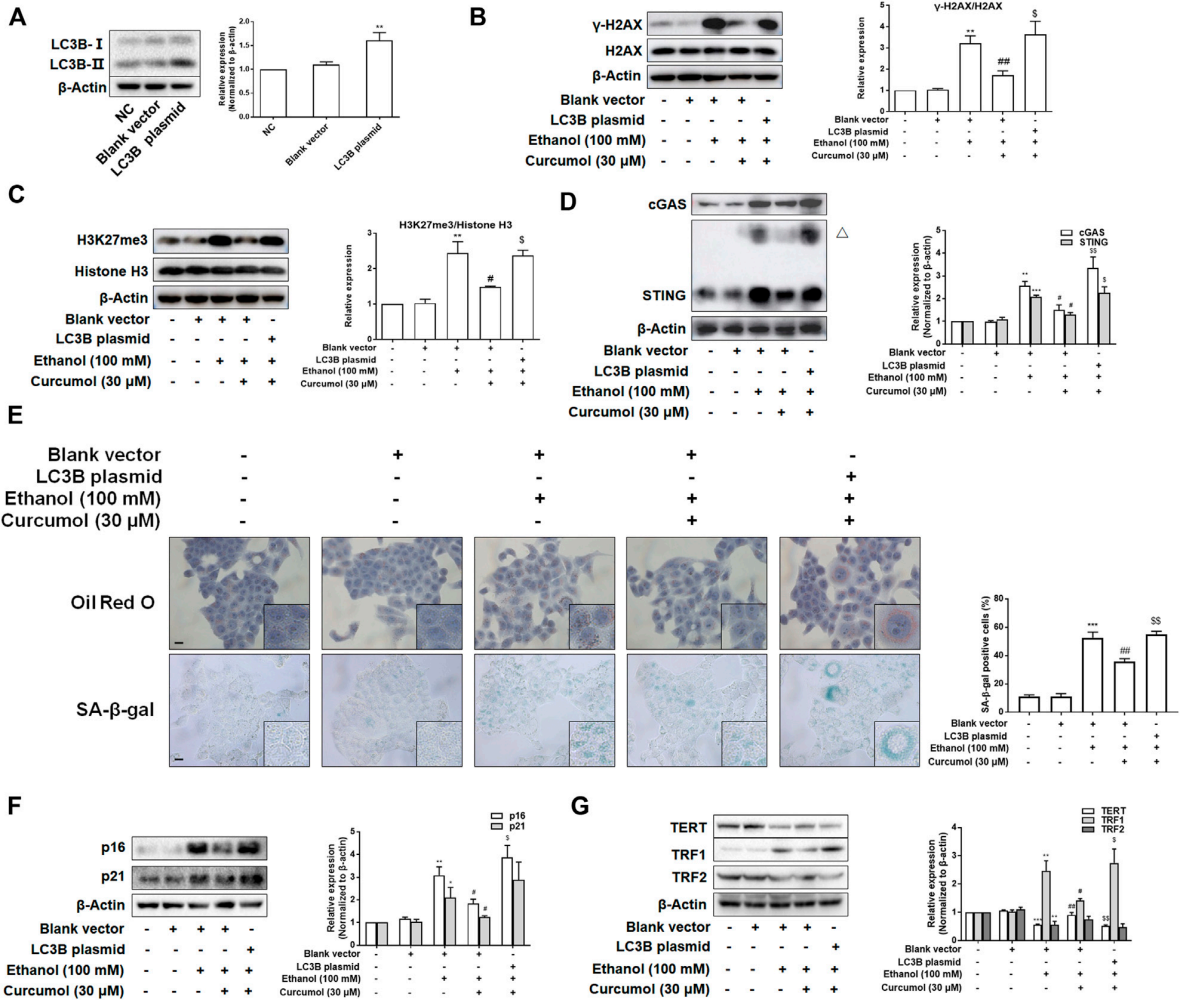
Overexpression of LC3B attenuated curcumol’s effect on ethanol-induced cellular senescence in LO2 cells. **(A–D)** Western blot analyses of the transfection efficiency of LC3B plasmid and the expression of γ-H2AX, H3K27me3, cGAS, and STING in LO2 cells, *n* = 3. STING blots were performed under non-reducing conditions. △ STING dimer. Data are represented as mean ± SD. Significance: **p < 0.01 and ***p < 0.001 versus blank vector group, ^#^ *p* < 0.05 and ^##^ *p* < 0.01 versus blank vector + ethanol group, ^$^ *p* < 0.05 and ^$$^ *p* < 0.01 versus blank vector + ethanol + curcumol group. **(E)** Representative images of oil red O staining and SA-β-gal staining (scale bar, 100 μm) in LO2 cells, *n* = 3. Data are represented as mean ± SD. Significance: ***p < 0.001 versus blank vector group, ^##^ *p* < 0.01 versus blank vector + ethanol group, ^$$^ *p* < 0.01 versus blank vector + ethanol + curcumol group. **(F** and **G)** Western blot analyses of protein expression of senescence markers (p16 and p21) and telomere and telomerase related factors (TERT, TRF1, and TRF2) protein levels in LO2 cells, *n* = 3. Data are represented as mean ± SD. Significance: *p < 0.05, **p < 0.01, and ^***^ *p* < 0.001 versus blank vector group, ^#^ *p* < 0.05 and ^##^ *p* < 0.01 versus blank vector + ethanol group, ^$^ *p* < 0.05 and ^$$^ *p* < 0.01 versus blank vector + ethanol + curcumol group.

To test whether LC3B overexpression weakened the effects of curcumol on ethanol-induced CCF formation and cellular senescence, we transfected LO2 cells with LC3B plasmid and the transfection efficiency was confirmed by western blot (Figure 6A). The results showed that overexpression of LC3B abolished the effect of curcumol on ethanol-induced CCF formation (Figures 6B and C) and cGAS-STING pathway activation (Figure 6D). Curcumol’s inhibition on lipid accumulation, cellular senescence, and senescence-related factors expression was also neutralized in cells transfected with LC3B plasmid (Figures 6E–G). These findings provided further support for the notion that curcumol’s inhibition of hepatocyte senescence was derived from suppression of LC3B expression and subsequent LC3B-lamin B1 interaction in AFLD.

### Curcumol Ameliorates Lipid Accumulation and Cellular Senescence in AFLD Through Blocking the Interaction of LC3B–Lamin B1 and Subsequent Inhibition of the CCF-cGAS-STING Pathway

**FIGURE 7:**
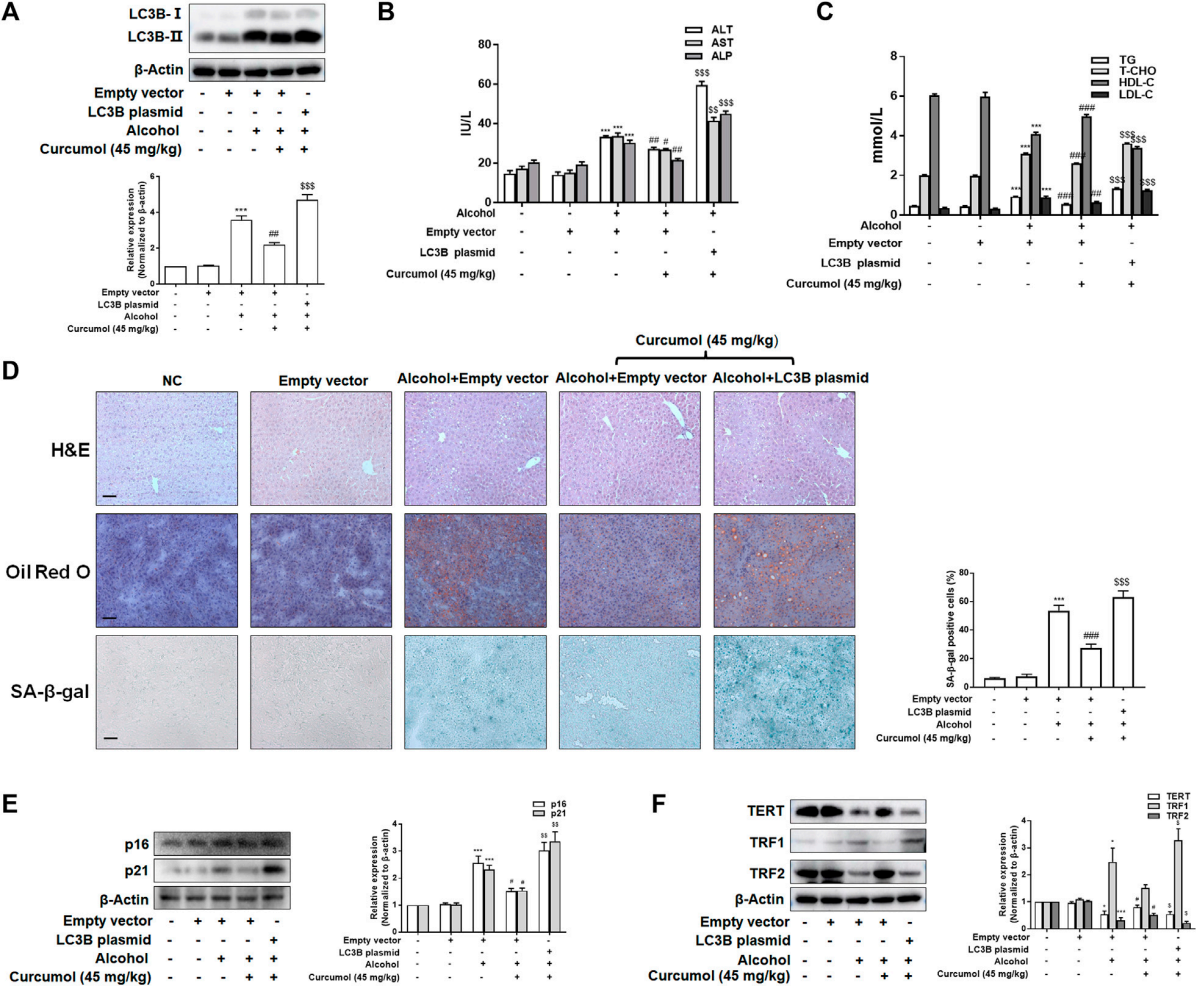
Curcumol’s repression of the interaction of LC3B-lamin B1, which ameliorates lipid accumulation and cellular senescence in the liver of AFLD. **(A)** Western blot analyses of the transfection efficiency of LC3B overexpression plasmid, *n* = 3. Data are represented as mean ± SD. Significance: ***p < 0.001 versus empty vector group, ^##^ *p* < 0.01 versus empty vector + ethanol group, ^$$$^ *p* < 0.001 versus empty vector + ethanol + curcumol group. **(B** and **C)** Determination of serum ALT, AST, ALP, T-CHO, TG, LDL-C, and HDL-C levels, *n* = 5. Data are represented as mean ± SD. Significance: ***p < 0.001 versus empty vector group; ^#^ *p* < 0.05, ^##^ *p* < 0.01, ^###^ *p* < 0.001 versus alcohol + empty vector group; ^$$^ *p* < 0.01, ^$$$^ *p* < 0.001 versus alcohol + empty vector + curcumol (45 mg/kg) group. **(D)** Liver sections were stained with H&E, oil red O staining, and SA-β-gal staining. Scale bars, 100 μm. Data are represented as mean ± SD. Significance: ***p < 0.001 versus empty vector group, ^###^ *p* < 0.001 versus empty vector + alcohol group, ^$$$^ *p* < 0.001 versus empty vector + alcohol + curcumol group. **(E** and **F)** Western blot analyses of senescence markers (p16 and p21) and telomere and telomerase related factors (TERT, TRF1, and TRF2) protein levels in the liver tissues, *n* = 3. Data are represented as mean ± SD. Significance: *p < 0.05 and ***p < 0.001 versus empty vector group, ^#^ *p* < 0.05 versus empty vector + alcohol group, ^$^ *p* < 0.05 and ^$$^ *p* < 0.01 versus empty vector + alcohol + curcumol group.

**FIGURE 8:**
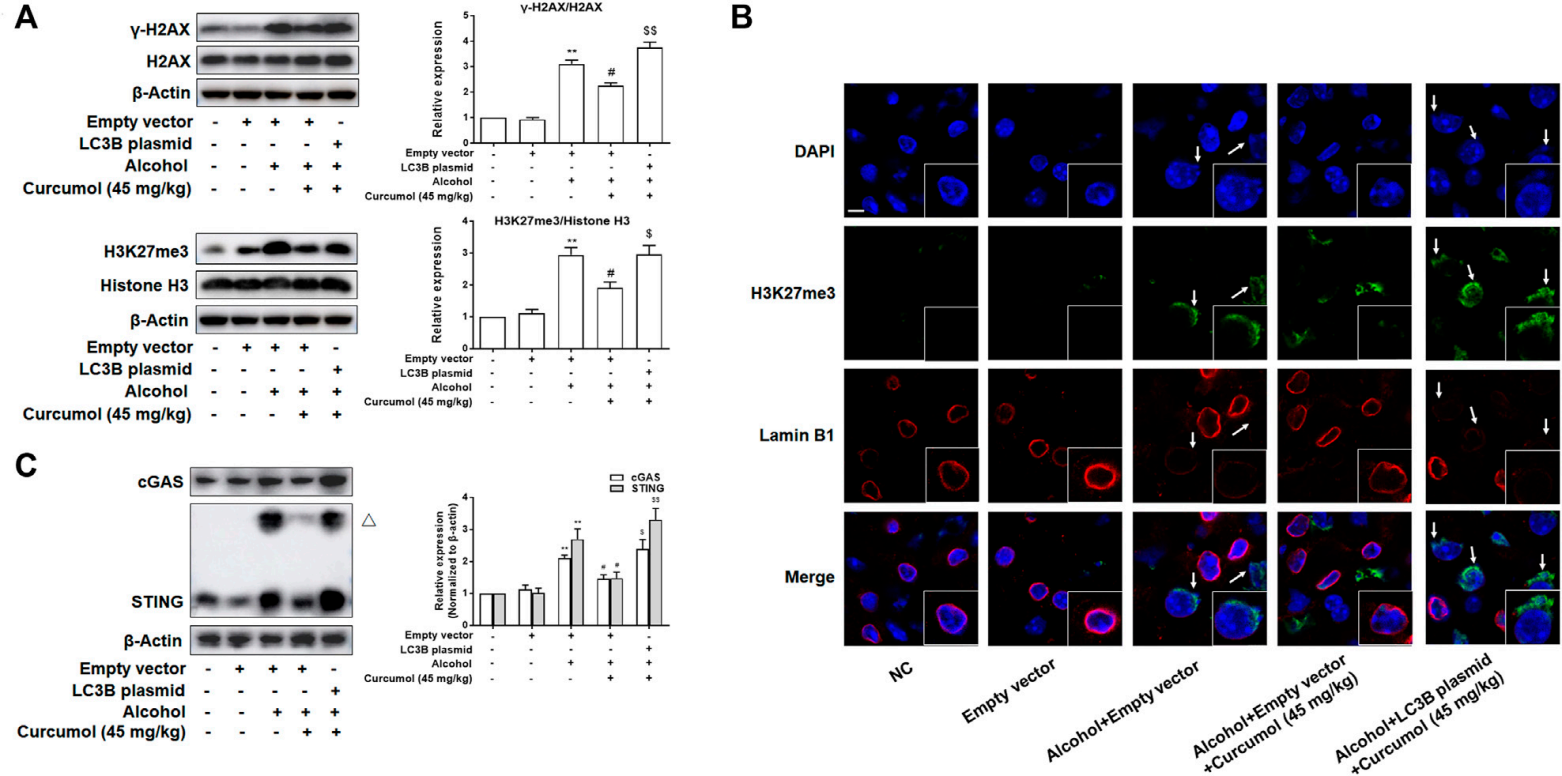
Curcumol’s repression of the interaction of LC3B-lamin B1, which inhibited the formation of CCF and the activation of cGAS-STING in the liver of AFLD. **(A)** Western blot analyses of the classical markers of CCF (γ-H2AX and H3K27me3) protein levels in the liver tissues, *n* = 3. Data are represented as mean ± SD. Significance: **p < 0.01 versus empty vector group, ^#^ *p* < 0.05 versus empty vector + alcohol group, ^$^ *p* < 0.05 and ^$$^ *p* < 0.01 versus empty vector + alcohol + curcumol group. **(B)** Immunofluorescence staining analyses of the production of CCF by detecting the expressions of lamin B1 and H3K27me3. CCF (green) and the loss of Lamin B1 (red) are indicated by arrows. *n* = 3. Sacle bar, 7.5 μm. **(C)** Western blot analyses of the expression of cGAS and STING in the liver tissues, *n* = 3. STING blots were performed under non-reducing conditions. △ STING dimer. Data are represented as mean ± SD. Significance: **p < 0.01 versus empty vector group, ^#^ *p* < 0.05 versus empty vector + alcohol group, ^$^ *p* < 0.05 and ^$$^ *p* < 0.01 versus empty vector + alcohol + curcumol group.

We finally attempted to confirm the abovementioned findings in ethanol liquid diet-fed C57BL/6J mice with lentivirus-packaged LC3B overexpression plasmids injected into the tail vein. The results showed that overexpression of LC3B significantly counteracted the ameliorative effect of curcumol on alcohol-induced liver damage, lipid metabolism disorder, and hepatic lipid accumulation (Figures 7A–D). Curcumol’s effects on hepatocyte senescence and senescence-related factors and telomere/telomerase system were also offset by transfection with LC3B plasmid (Figures 7D–F). Consistent with these results, overexpression of LC3B led to a significant neutralization of curcumol’s effect on CCF formation and cGAS-STING pathway activation (Figures 8A–C). These findings provided direct evidence that curcumol could ameliorate AFLD through blocking LC3B–lamin B1 interaction and subsequent inhibition of CCF-cGAS-STING pathway-mediated hepatocyte senescence.

## Discussion

Hepatocyte senescence correlated closely with the occurrence and development of alcoholic liver disease, and the inhibition of hepatocyte senescence could improve alcoholic liver disease (Aravinthan et al., 2013; Kong et al., 2021). However, there are no in-depth reports on the relationship between cellular senescence and AFLD. In this study, we observed that curcumol inhibited the senescence of hepatocytes in AFLD, as manifested by regulation of the major hallmarks of cellular senescence: SA-β-gal activity, the expression of p16^Ink4a^, p21, and telomere/telomerase system-related factors (Hernandez-Segura et al., 2018). Telomeric repeat binding factors 1 (TRF1) negatively regulates telomere length, while telomeric repeat binding factors 2 (TRF2) can delay senescence by protecting critically short telomeres from fusion (Karlseder et al., 2002; Azarm et al., 2020). This observation provoked us to investigate the molecular mechanisms for curcumol’s anti-senescence in AFLD. Recent studies have shown that the chromatin is reorganized and degraded in the nucleus when cells encounter stressful stimuli, including alcohol exposure (Chen et al., 2011; Petitpas et al., 2013; Bhatia et al., 2019). Then, the nuclear vesicles containing chromatin fragments appeared and were modified and transferred into the cytoplasm to become CCF (Ivanov et al., 2013). CCF are derived from heterochromatin regions of transcriptional inhibition and contain genomic DNA (γ-H2AX) and heterochromatin markers H3K9me3 and H3K27me3 (Ivanov et al., 2013; Dou et al., 2015). The formation of CCF could promote the secretion of SASP factors, such as chemokines, colony stimulating factors, and related interleukins, which affect the cell microenvironment and accelerate self-senescence and induce senescence of adjacent cells (Dou et al., 2017). In the current study, our data showed that curcumol abrogated the effect of ethanol on the expression of γ-H2AX and H3K27me3, suggesting that curcumol’s suppression of hepatocyte senescence may be related to the inhibition of CCF formation. In addition, we found that ethanol elevated the secretion of SASP-related inflammatory cytokines, which was considerably diminished by curcumol. However, exposure to ethanol for 24 h induced a relatively lower increase in the expression of inflammatory factors in LO2 cells than in some other reports (Dou et al., 2017; Takahashi et al., 2018). The reason for this discordance might be that the ethanol exposure time used in this study was relatively short so that only part of the cells entered senescence. It is also likely that most of the senescent cells were at an early stage of senescence and did not reach their peak level of secretion. Nevertheless, this relatively low extent of increase in ethanol-induced inflammatory cytokines in LO2 cells was in accordance with those of the *in vivo* experiment and some previous reports (Herranz et al., 2015; Singh et al., 2020; Jiang et al., 2021), suggesting that exposure to ethanol for 24 h might induce a low level of SASP-related factors in LO2 cells. Taken together, these results indicated that curcumol’s inhibition of CCF formation might reduce the secretion of SASP-related inflammatory factors and further depressed the senescence of hepatocytes in AFLD.

We further investigated the mechanisms underlying CCF regulation of hepatocyte senescence and identified a key role for cGAS-STING in this context. cGAS-STING mainly recognized the cytoplasmic DNA produced by pathogen infection (Sun et al., 2013; Wu et al., 2013). The activation of cGAS-STING could inhibit microbial infection and inflammation (Ishikawa and Barber, 2008; Ishikawa et al., 2009; Barber, 2015). A recent study reported that CCF could be recognized by cGAS in senescent cells, and the activation of cGAS-STING could promote the massive secretion of SASP (Gluck et al., 2017). In this study, curcumol inhibited the effects of ethanol on cGAS and STING expression and the activation of STING, including the formation of homo-dimers and redistribution into aggregates. These results led us to study whether cGAS-STING mediated the regulatory effect of curcumol on ethanol-induced hepatocyte senescence. Herein, overexpression of cGAS attenuated the inhibition effect of curcumol on cellular senescence as manifested by its influence on senescence-related factors and the telomere/telomerase system. Therefore, the inactivation effect of curcumol on cGAS-STING may be a key signal pathway between CCF and hepatocyte senescence in AFLD. In addition, we could not exclude the possibility that the cytoplasmic chromatin fragment originates from disrupted micronuclei. It has been reported that DNA and chromatin fragments leaking from disrupted micronuclei triggered the innate immune cGAS-STING mechanism that promoted inflammation which could cause a wide range of age-related diseases (Hatch et al., 2013; Guo et al., 2019; Krupina et al., 2021). Disrupted micronuclei are characterized by nuclear membrane collapse, mainly caused by the degradation of Lamin B1 (Hatch et al., 2013). Moreover, micronuclei also contained γ-H2AX and H3K27me3 proteins (Terradas et al., 2009; Postberg et al., 2019). Therefore, it is not easy to distinguish whether chromatin fragments in cytoplasm come from the main nucleus with a damaged nuclear membrane or disrupted micronuclei.

Based on the experimental observations, we conjectured that the inactivation of cGAS-STING, the inhibition of SASP-related inflammatory factors secretion, and subsequent cellular senescence could be due to the effect of curcumol on the formation of CCF. So, what is the reason why curcumol inhibits the formation of CCF in AFLD? Recent studies have demonstrated that CCF is transferred from the nucleus into the cytoplasm due to the downregulation of the nuclear lamina protein lamin B1 and the loss of nuclear membrane integrity (Ivanov et al., 2013; Galluzzi et al., 2016). The autophagy protein LC3B directly interacts with lamin B1 in the nucleus, which induces lamin B1 loss and enhances oncogene-induced senescence in primary human cells (Dou et al., 2015; Leidal and Debnath, 2015; Dou et al., 2016). Moreover, lamin B1 is closely related to cellular senescence (Shimi et al., 2011; Shah et al., 2013). Based on these findings, we further explored the impact of curcumol on the interaction of LC3B and lamin B1 in the models of AFLD. Our data showed that curcumol abrogated ethanol’s inhibitory effects on the expression of lamin B1. As expected, curcumol could significantly decrease LC3B levels concentration-dependently in ethanol-treated hepatocytes. Moreover, ethanol induced a large amount of LC3B-II accumulated in the nucleus, while curcumol inhibited the accumulation of LC3B-II and had a weak inhibitory effect on LC3B-I in the nucleus. Furthermore, ethanol enhanced the interaction of LC3B and lamin B1 and further promoted the loss of lamin B1 in hepatocytes. Combining these results, we speculated that curcumol’s inhibition of LC3B weakened the interaction of LC3B and lamin B1, which, in turn, reduced the degradation of lamin B1 in ethanol-treated hepatocytes.,

We further investigated the role of LC3B-lamin B1 interaction on curcumol’s suppression of CCF formation, cGAS-STING pathway, and cellular senescence. Overexpression of LC3B could competitively interrupt the effects of curcumol on the formation of CCF, the activation of cGAS-STING, and hepatocyte senescence in ethanol-treated hepatocytes. Furthermore, AFLD mice were transfected with lentivirus-packaged LC3B plasmid to establish the *in vivo* correlation. The results of *in vivo* experiments could provide consistent support for the prior *in vitro* findings. Importantly, these data further confirmed that curcumol’s inhibition of CCF-cGAS-STING and attenuation of cellular senescence might be dependent on the suppression of LC3B-lamin B1 interaction.

AFLD is characterized by lipid accumulation and can progress to alcoholic steatohepatitis, fibrosis, cirrhosis, and cancer in the liver (Koelmel et al., 2022). Our results showed that curcumol could inhibit ethanol-induced lipid accumulation to improve liver steatosis. So, does curcumol’s inhibition of ethanol-induced cellular senescence also affect lipid accumulation in hepatocytes? Although it has been reported that hepatocyte senescence could drive hepatic steatosis and elimination of senescent cells was able to reduce steatosis in NAFLD (Ogrodnik et al., 2017; Ogrodnik and Jurk, 2017; Papatheodoridi et al., 2020), the exact mechanism is not clear in AFLD. Some studies revealed that the secretion of some SASP-related inflammatory factors could affect lipid accumulation (Kim et al., 2020; Wang et al., 2020). For example, inflammatory cytokine CCL2 could exacerbate hepatic steatosis in chronic hepatic injury (Baeck et al., 2012). We speculated that suppression of SASP-related inflammatory factors secretion might be responsible for curcumol’s inhibition of hepatic steatosis in AFLD. However, we could not provide direct evidence for the causation between hepatocyte senescence and hepatic steatosis. We will further verify this speculation through experiments in the future.

In summary, our data elucidated that curcumol’s suppression of hepatocyte senescence was associated with inhibition of CCF formation and subsequent inactivation of cGAS-STING in hepatocytes of AFLD. Furthermore, curcumol might inhibit the interaction of LC3B-lamin B1, which would benefit to reduce the formation of CCF. Our data elucidated the mechanisms underlying curcumol’s anti-AFLD activity and indicated a therapeutic target for alleviation of ethanol-induced hepatocyte senescence.

## Data Availability

The original contributions presented in the study are included in the article/Supplementary Material; further inquiries can be directed to the corresponding authors.

## Abbreviations

AFLD, alcoholic fatty liver disease; ALP, alkaline phosphatase; ALT, alanine aminotransferase; AST, aspartate transaminase; CCF, cytoplasmic chromatin fragments; CCK-8, cell counting Kit-8; CCL2, C-C motif chemokine ligand 2; cGAS, cyclic GMP-AMP synthase; CSF2, colony-stimulating factor 2; EdU, 5-ethynyl-2′-deoxyuridine; GAPDH, glyceraldehyde phosphate dehydrogenase; H&E, hematoxylin and eosin; H3K27me3, trimethylation of lysine 27 at histone H3; HDL-C, high-density lipoprotein cholesterol; IL-6, interleukin 6; LC3B, microtubule-associated protein 1 light chain 3 beta; LDL-C, low-density lipoprotein cholesterol; PBS, phosphate-buffered saline; RQ-PCR, real-time quantitative polymerase chain reaction; SA-β-gal, senescence-associated β-galactosidase; SASP, senescence-associated secretory phenotype; STING, stimulator of interferon genes; T-CHO, total cholesterol; TERT, telomerase reverse transcriptase; TG, triglyceride; TRF1, telomeric repeat binding factors 1; TRF2, telomeric repeat binding factors 2; VEGF, vascular endothelial growth factor; γ-H2AX, phosphorylation of histone H2AX, gamma-H2AX.
